# Doxycycline exposure during adolescence and future risk of non-affective psychosis and bipolar disorder: a total population cohort study

**DOI:** 10.1038/s41398-021-01574-6

**Published:** 2021-09-08

**Authors:** Fredrik Upmark, Hugo Sjöqvist, Joseph F. Hayes, Christina Dalman, Håkan Karlsson

**Affiliations:** 1 Department of Global Public Health, Karolinska Institutet, Stockholm, Sweden; 2 Division of Psychiatry, Faculty of Brain Sciences, University College London, London, UK; 3 Centre for Epidemiology and Social Medicine, The Region Stockholm, Stockholm, Sweden; 4 Department of Neuroscience, Karolinska Institutet, Stockholm, Sweden

**Keywords:** Pathogenesis, Schizophrenia, Bipolar disorder, Clinical pharmacology

## Abstract

Doxycycline has been hypothesized to prevent development of severe mental illness (SMI) through the suppression of microglia, especially if administered during the intense synaptic pruning period of adolescence. However, results from register studies on potential benefits differ considerably. The aim of the present study was to determine whether doxycycline exposure during adolescence is associated with reduced SMI risk, and to investigate if a direct and specific causality is plausible. This is a Swedish national population register-based cohort study of all individuals born from 1993 to 1997, followed from the age of 13 until end of study at the end of 2016. The primary exposure was cumulative doxycycline prescription ≥3000 mg and outcomes were first diagnosis of non-affective psychosis (F20–F29) and first diagnosis of bipolar disorder (F30–F31). Causal effects were explored through Cox regressions with relevant covariates and secondary analyses of multilevel exposure and comparison to other antibiotics. We found no association between doxycycline exposure and risk of subsequent non-affective psychosis (adjusted hazard ratio (HR) 1.15, 95% CI 0.73–1.81, *p* = 0.541) and an increased risk of subsequent bipolar disorder (adjusted HR 1.95, 95% CI 1.49–2.55, *p* < 0.001). We do not believe the association between doxycycline and bipolar disorder is causal as similar associations were observed for other common antibiotics.

## Introduction

Severe mental illness (SMI) often results in lifelong and extensive consequences for individuals and society, whereof most evident is a reduced life expectancy in the magnitude of decades [1–5]. Recent studies suggest that individuals with SMI have not benefited from the same increase in life expectancy as the general population [6, 7]. Thus, the substantial gap in mortality, a major indicator of a disadvantaged life, is not closing but rather widening. There are no current interventions that prevent or delay SMI onset.

Schizophrenia and bipolar disorder are two SMIs that exhibit substantial overlap in terms of polygenetic risk and brain structural abnormalities [8, 9]. Both illnesses are associated with reductions in cortical thickness and subcortical volumes [10, 11]. Recent studies indicate that microglia may play important roles in the pathogenesis of SMI [12]. Results from several lines of research support the hypothesis that excessive activity in the removal (pruning) of synapses by microglia that normally occurs during adolescence contributes to the onset of schizophrenia in early adulthood [13–18]. Two substances known to suppress microglia activation, in both experimental in vivo settings and in vitro [19, 20], are the chemically closely related tetracycline antibiotics minocycline and doxycycline with comparable abilities to cross the blood–brain barrier [21–23]. Recently, Sellgren et al. reported that minocycline indeed reduced microglia-mediated synapse uptake in vitro [24]. Moreover, Sellgren et al. added analyses of 22,027 electronic health records from two large US medical centers and found an inverse association (HR 0.58, 95% confidence interval (CI) 0.39–0.88) between minocycline and/or doxycycline exposure during adolescence for a minimum of 90 days (3811 individuals) and risk of later diagnosis of non-affective psychosis, including schizophrenia (F20–F29) [24]. This contrasts an earlier electronic healthcare register study from UK by Herrero-Zazo et al. who compared 13,248 minocycline exposed for a minimum of 42 days with 14,393 matched controls and found no preventive effect on SMI from early minocycline prescription [25]. Comparing exposure between these two studies is not entirely trivial. Minocycline and doxycycline are slightly different substances. In addition, days of exposure cannot be directly translated to exposure in mg as daily dosage for long-term treatment of acne varies. However, 50–100 mg/day is a common acne dosage for both minocycline and doxycycline [26, 27]. If a typical dosage of 100 mg/day is used for conversion, then the Sellgren et al. threshold would roughly translate to a total exposure of ≥9000 mg and the Herrero-Zazo et al. to ≥4200 mg.

A concern has recently been raised that results from the above studies may be confounded by acne, a condition sometimes treated with long-term prescriptions of minocycline and/or doxycycline [28].

Another potential confounder is isotretinoin exposure, a nonantibiotic acne drug which has been hypothesized to increase the risk of SMI [29, 30]. However, confounding by isotretinoin was addressed by both Herrero-Zazo et al. [25] who excluded individuals from the study if exposed to isotretinoin prior to baseline and censored if exposure occurred during follow-up, and Sellgren et al. [24] who reported unchanged results in a sensitivity analysis after exclusion of all isotretinoin exposed individuals. A third potential source of confounding is through unmeasured socioeconomic and cultural factors, which might affect both healthcare utilization, including seeking help for acne, and the risk of later SMI.

The primary aim of the present study was to use Swedish national population registers to investigate if doxycycline exposure during adolescence (minocycline not being prescribed in Sweden) was associated with subsequent non-affective psychosis (ICD-10 F20–F29) or bipolar disorder (ICD-10 F30–F31) diagnoses. The secondary aim was to investigate if potentially observed associations were specific to doxycycline, as compared to other antibiotics, i.e., if a direct and specific causality was plausible. In addition, we performed sensitivity analyses to address possible confounding by acne and/or isotretinoin.

## Methods

### Study design and population

The study, approved by the regional ethics committee (registration number 2010-1185-31-5), was based on a cohort of all individuals born between Jan 1, 1993 and Dec 31, 1997, who were Swedish residents at the age of 13, with no previous record of non-affective psychosis or bipolar disorder (ICD-10 F20–F31). The cohort as well as data on exposures, outcomes and covariates was extracted from the record linkage Psychiatry Sweden, specifically developed for studies of mental health, linking Swedish national and regional administrative registers, using the unique national identification number assigned to Swedish citizens at birth and to immigrants upon arrival in the country [31].

- The Total Population Register: demographic data on Swedish residents, full coverage on vital status and >90% coverage on migration information [32].
- The Multi-Generation Register: linking people to their biological or adoptive parents, near full coverage [33].
- The Longitudinal Integration Database for Health Insurance and Labor Market Studies: containing data on socioeconomic variables for people aged 16 years and older since 1990, estimated coverage >95% [34].
- The National Patient Register: includes records of all inpatient care in Sweden, complete coverage for psychiatric disorders since 1973 and somatic disorders since 1987. Also includes records on specialized outpatient care visits since 2001 [35].
- The National Prescribed Drug Register: contains data on all prescriptions since July 1, 2005, excluding over-the-counter medications and drugs used in hospitals [36].

#### Primary exposure

A binary exposure of a cumulative dispensed doxycycline ≥3000 mg, equal to three typical doxycycline prescriptions of 10 days × 100 mg.

#### Primary outcomes

First diagnosis of non-affective psychosis (F20–F29) or bipolar disorder (F30–F31) in inpatient or outpatient record.

#### Covariates

Regressions were performed as univariable analyses (crude estimates)—without stratification and without the use of covariates—and as multivariable analyses (adjusted estimates) stratified by sex and controlling for the following set of a priori selected covariates: year of birth, Swedish healthcare region (21 categories), origin of the individual (Sweden, South and Central America, Eastern Europe and Russia, sub-Saharan Africa, other international region), origin of parents (Sweden, one parent not from Sweden or both parents not from Sweden), highest completed education among parents (less than elementary school, elementary school, upper secondary school ≤2 years, upper secondary school 3 years, postsecondary education <3 years, postsecondary education ≥3 years, postgraduate education), income of parents (five levels), one or both parents diagnosed with non-affective psychosis and finally one or both parents diagnosed with bipolar disorder. Covariates were measured at baseline, i.e., when the subjects turned 13. Some of the categorical covariates had a minor proportion of missing values, which for each of these covariates was coded as an additional missing category.

#### Statistical analyses

Analyses were performed as Cox regressions with each analysis run twice, first with outcome defined as non-affective psychosis and then as first diagnosis of bipolar disorder. Age was used as the time scale and all subjects entered the risk pool at the age of 13 (i.e., no delayed entries). Exposure to prescribed doxycycline and other investigated drugs was measured from the age of 13 onward. Exposed individuals’ time at risk was split in time as unexposed (until reaching the cumulative exposure threshold) and time as exposed (after reaching the threshold). A delay window of 6 months was applied between the date a drug was dispensed and effective exposure reclassification. Subjects were followed until one of the following whichever occurred first: record of defined outcome, end of study period at Dec 31, 2016 (between the age of 19 and 24), or right censoring at emigration or death before end of study period. The proportional hazard assumption of Cox regressions was assessed through tests of nonzero slope of scaled Schoenfeld residuals [37].

### Secondary analyses

#### Doxycycline, thresholds of exposure

To investigate different levels of cumulative doxycycline exposure, additional analyses were run simultaneously estimating five intervals of exposure: 1000–1999, 2000–2999, 3000–4999, 5000–9999, and ≥10,000 mg, i.e., thresholds equivalent to exposures of 1, 2, 3, 5, and 10 typical prescriptions.

#### Non-doxycycline antibiotics, binary exposure

As comparison to doxycycline, exposure estimates for the following groups of 20 commonly prescribed antibiotics were calculated: cefalosporines (cefadroxil), fluoroquinolones (ciprofloxacin, levofloxacin, moxifloxacin, and norfloxacin), macrolides and lincosamides (azithromycin, clarithromycin, clindamycin, erythromycin, and roxithromycin), nitrofurantoin (nitrofurantoin), penicillins (amoxicillin, amoxicillin + enzyme inhibitor, flucloxacillin, phenoxymethylpenicillin [penicillin V], and pivmecillinam), tetracyclines (lymecycline and tetracycline), and trimethoprim (trimethoprim and trimethoprim + sulfamethoxazol), see Supplementary Table 1 for corresponding ATC codes. To provide some degree of comparability between antibiotics, exposure was measured in number of standard treatments. The size of a standard treatment was set to the typical prescribed amount for each medication, with typical values identified from the distribution of studied data. Threshold levels of cumulative exposure (in number of standard treatments) were set by rounding the square root of number of antibiotics within each group, resulting in thresholds roughly reflecting an assumed all other things equal increased probability of higher total exposure within larger groups.

#### Non-doxycycline antibiotics, thresholds of exposure

To investigate different levels of exposure, an analysis was run estimating five intervals of cumulative non-doxycycline antibiotic exposure with thresholds of 1, 2, 3, 5, and 10 standard treatments.

### Sensitivity analyses

For each primary Cox regression, a set of corresponding sensitivity analyses were run:

1. Stratifying by gender to compare the results for women and men.
2. Different estimates within different age-spans. Five age intervals were defined by quintiles of age at event, i.e., with an approximately equal number of non-affective psychosis/bipolar disorder diagnoses within each span.
3. A cross-sectional approach using logistic regression. Exposure was measured from the age of 13–20, baseline set to 20 and follow-up at the age of 22 (i.e., only including individuals with data up until 22 and no recorded outcome before 20). Binary exposure threshold was set to ≥2000 mg of doxycycline to roughly reflect the cross-sectional approach’s more limited time of exposure.
4. To investigate potential confounding by acne, analyses were performed excluding all individuals with a documented acne diagnosis before or during the study period.
5. Finally, to investigate potential associations to the nonantibiotic acne drug isotretinoin, analyses were performed on all individuals but with isotretinoin as exposure (instead of doxycycline). Binary exposure threshold was set to ≥3000 mg, covering most individuals with >0 mg of dispensed isotretinoin. (A total of 3000 mg being the same threshold as for doxycycline exposure in primary analysis was a mere coincidence.)

## Results

The study group fulfilling the inclusion criteria of being born between Jan 1, 1993 and Dec 31, 1997, being Swedish residents at the age of 13, and with no previous record of non-affective psychosis or bipolar diagnosis consisted of 541,940 individuals, of whom 264,627 were female (49%) and 277,313 male (51%). Average follow-up time was 8.5 years. During follow-up 2182 (0.4%) of the individuals had a first diagnosis of non-affective psychosis, 3367 (0.6%) a first diagnosis of bipolar disorder and 7043 (1.3%) reached the primary exposure of ≥3000 mg dispensed doxycycline. See Supplementary Tables 2–4 for the distributions of primary exposure and outcomes across covariates.

Gender differences, primarily regarding typical onset age of non-affective psychosis (including schizophrenia), were a priori assumed to violate the proportional hazard assumption of Cox regressions. Support for the assumed violation was calculated for non-affective psychosis (F20–F29) and bipolar disorder (F30–F31) with significant results (*p* values of 0.0002 and 0.0245, respectively). After stratification by sex none of the regressions violated the proportional hazards assumption.

### Primary exposure

The crude hazard ratio (HR) from the binary exposure analysis showed no evidence of a relationship between doxycycline and risk of subsequent non-affective psychosis (HR 1.08, 95% CI 0.69–1.70, *p* = 0.728), which was consistent with pooled estimates from the analysis stratified by sex and adjusted for covariates (HR 1.15, 95% CI 0.73–1.81, *p* = 0.541), see Table 1. Schizophrenia (F20) estimates were calculated separately and resulted in no difference to all non-affective psychosis (F20–F29), data not shown.

**Table 1:** Hazard ratios^a^ (HRs) of binary doxycycline exposure (*n* = 541 940).

	Univariable Cox regression, without stratification and without covariates			Multivariable Cox regression, stratified by sex and with covariates		
Outcome/exposure	HR	(95% CI)		HR	(95% CI)	
Non-affective psychosis (ICD-10 F20–F29)						
Doxycycline ≥3000 mg	1.08	(0.69–1.70)	*p* = 0.728	1.15	(0.73–1.81)	*p* = 0.541
Bipolar disorder (ICD-10 F30–F31)						
Doxycycline ≥3000 mg	2.10	(1.60–2.74)	*p* < 0.001	1.95	(1.49–2.55)	*p* < 0.001

^a^HRs relative to base category of 0–2999 mg.

The crude binary exposure analysis showed a significant association between doxycycline and increased risk of subsequent bipolar disorder (HR 2.10, 95% CI 1.60–2.74, *p* < 0.001). The association remained in the fully adjusted model (HR 1.95, 95% CI 1.49–2.55, *p* < 0.001), see Table 1.

### Secondary analyses

#### Doxycycline, thresholds of exposure

Results from interval analyses were consistent with the binary results. For non-affective psychosis, the overall picture was of no association. For bipolar diagnosis, the estimates exhibited a dose–response relationship with higher levels of exposure associated with higher risk of subsequent diagnosis, see Table 2.

**Table 2:** Hazard ratios^a^ (HRs), thresholds of doxycycline exposure (*n* = 541,940).

	Univariable Cox regression, without stratification and without covariates			Multivariable Cox regression, stratified by sex and with covariates		
Outcome/exposure	HR	(95% CI)		HR	(95% CI)	
Non-affective psychosis (ICD-10 F20–F29)						
Doxycycline 1000–1999 mg	1.00	(0.82–1.22)	*p* = 0.995	1.09	(0.89–1.32)	*p* = 0.401
Doxycycline 2000–2999 mg	1.47	(1.00–2.17)	*p* = 0.051	1.65	(1.12–2.43)	*p* = 0.012
Doxycycline 3000–4999 mg	1.06	(0.60–1.87)	*p* = 0.839	1.14	(0.65–2.01)	*p* = 0.652
Doxycycline 5000–9999 mg	1.74	(0.78–3.87)	*p* = 0.177	1.90	(0.85–4.25)	*p* = 0.115
Doxycycline ≥10,000 mg	0.47	(0.07–3.36)	*p* = 0.453	0.48	(0.07–3.38)	*p* = 0.458
Bipolar disorder (ICD-10 F30–F31)						
Doxycycline 1000–1999 mg	1.83	(1.61–2.07)	*p* < 0.001	1.59	(1.40–1.80)	*p* < 0.001
Doxycycline 2000–2999 mg	2.00	(1.51–2.65)	*p* < 0.001	1.70	(1.28–2.25)	*p* < 0.001
Doxycycline 3000–4999 mg	2.09	(1.48–2.95)	*p* < 0.001	1.87	(1.33–2.64)	*p* < 0.001
Doxycycline 5000–9999 mg	2.30	(1.27–4.16)	*p* = 0.006	2.05	(1.13–3.70)	*p* = 0.018
Doxycycline ≥10,000 mg	2.03	(0.91–4.51)	*p* = 0.084	2.40	(1.08–5.35)	*p* = 0.032

^a^All HRs relative to base category of 0–999 mg.

#### Non-doxycycline antibiotics, binary exposure

Crude HRs for all non-doxycycline antibiotics showed a weak positive association between exposure and risk of subsequent non-affective psychosis (HR 1.16, 95% CI 1.02–1.32, *p* = 0.020), which was consistent with the regression stratified by sex and adjusted for covariates (HR 1.37, 95% CI 1.20–1.56, *p* < 0.001), see Table 3.

**Table 3:** Hazard ratios^a^ (HRs) of binary exposure of non-doxycycline antibiotics (*n* = 541 940).

	Number of substances within group	Exposure threshold (in nr. of standard treatments)	Univariable Cox regression, without stratification and without covariates			Multivariable Cox regression, stratified by sex and with covariates		
Outcome/exposure			HR	(95% CI)		HR	(95% CI)	
*Non-affective psychosis (ICD-10 F20–F29)*								
Cefalosporines (cefadroxil)	1	≥1	0.93	(0.75–1.16)	*p* = 0.541	1.07	(0.86–1.33)	*p* = 0.559
Fluoroquinolones	4	≥2	1.51	(0.84–2.74)	*p* = 0.171	1.66	(0.92–3.00)	*p* = 0.094
Macrolides and lincosamides	5	≥2	1.20	(0.92–1.56)	*p* = 0.172	1.31	(1.01–1.70)	*p* = 0.043
Nitrofurantoin	1	≥1	0.90	(0.74–1.10)	*p* = 0.301	1.29	(1.05–1.60)	*p* = 0.017
Penicillins	5	≥2	1.07	(0.95–1.19)	*p* = 0.257	1.22	(1.09–1.37)	*p* < 0.001
Tetracyclines (other than doxycycline)	2	≥1	1.14	(0.97–1.35)	*p* = 0.123	1.16	(0.98–1.37)	*p* = 0.086
Trimethoprim	2	≥1	1.37	(1.09–1.73)	*p* = 0.008	1.71	(1.35–2.16)	*p* < 0.001
All antibiotics								
All antibiotics (other than doxycycline)	20	≥4	1.16	(1.02–1.32)	*p* = 0.020	1.37	(1.20–1.56)	*p* < 0.001
*Bipolar disorder (ICD-10 F30–F31)*								
Cefalosporines (cefadroxil)	1	≥1	1.36	(1.17–1.58)	*p* < 0.001	1.24	(1.06–1.44)	*p* *=* 0.006
Fluoroquinolones	4	≥2	2.24	(1.50–3.35)	*p* < 0.001	1.99	(1.33–2.97)	*p* *=* 0.001
Macrolides and lincosamides	5	≥2	1.50	(1.24–1.82)	*p* < 0.001	1.44	(1.19–1.74)	*p* < 0.001
Nitrofurantoin	1	≥1	2.30	(2.06–2.57)	*p* < 0.001	1.51	(1.35–1.70)	*p* < 0.001
Penicillins	5	≥2	1.96	(1.81–2.12)	*p* < 0.001	1.66	(1.53–1.79)	*p* < 0.001
Tetracyclines (other than doxycycline)	2	≥1	1.12	(0.98–1.29)	*p* *=* 0.095	1.19	(1.04–1.36)	*p* = 0.014
Trimethoprim	2	≥1	2.60	(2.25–2.99)	*p* < 0.001	1.85	(1.60–2.14)	*p* < 0.001
All antibiotics								
All antibiotics (other than doxycycline)	20	≥4	1.97	(1.81–2.16)	*p* < 0.001	1.65	(1.51–1.81)	*p* < 0.001

^a^HRs relative to base category of 0–[exposure threshold].

Crude risk estimates indicated a positive association between exposure and risk of subsequent bipolar diagnosis (HR 1.97, 95% CI 1.81–2.16, *p* < 0.001). A somewhat attenuated association was observed from the regression stratified by sex and adjusted for covariates (HR 1.65, 95% CI 1.51–1.81, *p* < 0.001). The findings were consistent with estimates from exposure to individual groups of non-doxycycline antibiotics, where all investigated groups were associated with an increased risk of subsequent bipolar diagnosis, see Table 3.

#### Non-doxycycline antibiotics, thresholds of exposure

The interval analysis was coherent with results from the binary analysis. Crude estimates showed no clear association to risk of subsequent non-affective psychosis. Adjusted estimates indicated a slightly increased risk, see Table 4.

**Table 4:** Hazard ratios^a^ (HRs), thresholds of exposure of non-doxycycline antibiotics (*n* = 541 940).

	Univariable Cox regression, without stratification and without covariates			Multivariable Cox regression, stratified by sex and with full set of covariates		
Outcome/exposure	HR	(95% CI)		HR	(95% CI)	
*Non-affective psychosis (ICD-10 F20–F29)*						
All antibiotics (non-doxycycline)						
1.00–1.99 standard treatments	1.06	(0.95–1.19)	*p* = 0.294	1.14	(1.02–1.28)	*p* *=* 0.025
2.00–2.99 standard treatments	0.97	(0.83–1.13)	*p* = 0.681	1.09	(0.94–1.27)	*p* = 0.266
3.00–4.99 standard treatments	1.19	(1.03–1.38)	*p* = 0.017	1.41	(1.22–1.64)	*p* < 0.001
5.00–9.99 standard treatments	1.06	(0.89–1.28)	*p* = 0.508	1.32	(1.10–1.59)	*p* = 0.003
≥10.00 standard treatments	1.19	(0.88–1.62)	*p* = 0.252	1.50	(1.02–1.09)	*p* = 0.005
*Bipolar disorder (ICD-10 F30–F31)*						
All antibiotics (non-doxycycline)						
1.00–1.99 standard treatments	1.45	(1.31–1.59)	*p* < 0.001	1.33	(1.21–1.47)	*p* < 0.001
2.00–2.99 standard treatments	2.02	(1.81–2.25)	*p* < 0.001	1.77	(1.58–1.97)	*p* < 0.001
3.00–4.99 standard treatments	2.11	(1.88–2.36)	*p* < 0.001	1.77	(1.58–1.98)	*p* < 0.001
5.00–9.99 standard treatments	2.78	(2.46–3.13)	*p* < 0.001	2.22	(1.97–2.51)	*p* < 0.001
≥10.00 standard treatments	2.95	(2.42–3.60)	*p* < 0.001	2.36	(1.93–2.88)	*p* < 0.001

^a^All HRs relative to base category of 0–0.99 standard treatments.

For bipolar disorder, all levels of non-doxycycline exposure were associated with an increased risk of subsequent diagnosis. Higher levels of exposure were associated with higher risks, i.e. a dose–response pattern similar to that observed for doxycycline exposure, see Table 4.

### Sensitivity analyses

None of the sensitivity analyses, for either non-affective psychosis or bipolar disorder, generated results inconsistent with results from the primary analysis.

For both non-affective psychosis and bipolar disorder, separate analyses by gender resulted in no differences between subgroups of women and men for both crude and adjusted estimates.

Calculated estimates within five age-spans, defined by quintiles of age at outcome, resulted in no evidence for differences between the spans using chi-squared tests. Also, no trends were observed for either outcome.

Point estimated odds ratios (ORs) from logistic regressions were similar to their HR counterparts from primary analyses, but with wider confidence intervals due to the smaller sample size. Neither crude nor adjusted estimates for either non-affective psychosis (crude OR 1.43, 95% CI 0.78–2.60, *p* = 0.246/adj. OR 1.66, 95% CI 0.91–3.03, *p* = 0.100) or bipolar disorder (crude OR 1.99, 95% CI 1.28–3.08, *p* = 0.002/adj. OR 1.77, 95% CI 1.14–2.75, *p* = 0.011) indicated deviation from the results of the primary analysis.

Excluding all individuals with a record of an acne diagnosis (n = 25 471) resulted in estimates close to those obtained in the primary analysis for both non-affective psychosis (crude HR 1.05, 95% CI 0.64–1.73, *p* = 0.833/adj. HR 1.13, 95% CI 0.69–1.85, *p* = 0.628) and bipolar disorder (crude HR 2.30, 95% CI 1.74–3.03, *p* < 0.001/adj. HR 2.10, 95% CI 1.59–2.78, *p* < 0.001). Acne patients only estimates were calculated separately and resulted in no difference, data not shown.

We found no evidence for an association between isotretinoin and either non-affective psychosis or bipolar disorder. For non-affective psychosis, crude estimates were weakly positive (HR 1.39, 95% CI 1.02–1.91, *p* = 0.038), while the corresponding adjusted estimates were non-significant (HR 1.33, 95% CI 0.97–1.82, *p* = 0.074). For bipolar disorder, neither crude (HR 0.97, 95% CI 0.72–1.32, *p* = 0.859) nor adjusted (HR 1.11, 95% CI 0.81–1.50, *p* = 0.519) estimates indicated a relation to isotretinoin exposure.

## Discussion

In the present study, we observed no association between prescribed doxycycline during adolescence and subsequent risk of non-affective psychosis. However, an association with higher risk of bipolar disorder was observed.

To our knowledge, only a few register studies have been conducted on the potential protective effects of the tetracyclines doxycycline and minocycline on subsequent SMI, and the results from these studies differ considerably.

Our results with regard to non-affective psychosis risk are in general agreement with Herrero-Zazo and colleagues [25], but contrast with the results of Sellgren et al. [24], which indicated a decreased risk of subsequent non-affective psychosis.

The association between doxycycline exposure and increased risk of bipolar disorder was an unexpected finding. Even though the relationship exhibited a classical dose–response pattern, we do not consider this association likely to be of a direct causal nature, as similar associations were observed for all other groups of common antibiotics. It should be noted that we also found a small association between exposure to non-doxycycline antibiotics and subsequent non-affective psychosis. Several interpretations of these findings are possible. Besides an assumed presence of complex and unmeasured confounding, one plausible explanation is that individuals with a high risk, or already close to diagnosis, may exhibit a less healthy lifestyle—including a higher healthcare utilization and higher exposure to antibiotics (i.e., reverse causation). Such a pattern of higher pre-diagnosis healthcare utilization is known for schizophrenia [38, 39]. It is however unknown to what extent the present study’s association between antibiotic exposure and bipolar disorder is the result of a higher overall healthcare utilization among individuals with subsequent bipolar disorder diagnosis. Other interpretations, not dismissed by the study results, include the potential causal association between bacterial infections and/or exposure of antibiotic substances in general and subsequent mental disorders [40–43]. Additional research, possibly including the longitudinal analyses of pre-diagnosis healthcare utilization patterns among individuals with subsequent bipolar diagnosis, is needed to make more conclusive statements regarding causality.

### Strengths and limitations

One of the challenges for the study design was the absence of a chronological separation between prescriptions of doxycycline and occurrences of defined outcomes. Therefore, the primary analysis used Cox regression in combination with a continuously measured cumulative exposure with reclassification of subjects’ exposure status after passing set thresholds. The thresholds were selected from common levels of exposure among the subjects for both binary and multilevel analyses. A delay window of 6 months between the date a drug was dispensed and reclassification as exposed was applied with the intent to let the individuals fully consume the prescriptions before being reclassified, and to reduce potential reverse causation close to an upcoming diagnosis of SMI. An alternative approach would have been to measure exposure until a fixed age and then ignore further exposure during follow-up. This was done in the cross-sectional sensitivity analysis and resulted in point estimates close to the primary approach.

The cohort consisted of full population data from Swedish national registers, assuring close to full coverage and reliable data. Missing/unknown values occurred exclusively among four categorical covariates: origin of the individual (i.e., country of origin), Swedish healthcare region, highest completed education among parents, and income of parents. Occurrences of missing values were assumed to not primarily represent unknown information, but rather in themselves indicate a potential difference in relation to the main population. For example, missing values of income of parents were associated with a higher incidence of studied outcomes than any observed category of actual income, i.e., the fact that the values were missing seemed to provide more information than possible even from a perfect imputation, see Supplementary Tables 3 and 4. Therefore, for each of the affected covariates missing values were coded as an additional missing category.

The National Patient Register has almost a complete coverage of inpatient care from 1973. However, outpatient specialized care was not sufficiently covered until 2005, which is also valid for the National Prescribed Drug Register. Thus, the decision at which age to include subjects had to be chosen as a trade-off between maximizing time of follow-up and minimizing unknown drug exposure or diagnosis before time of inclusion. The age of 13, i.e., individuals born 1993 or later, was chosen taking into account that doxycycline in Sweden only rarely is prescribed before age 13. The inclusion age had an additional benefit in that studied outcomes (F20–F31) seldom occur before this age, resulting in few individuals being excluded due to a documented diagnosis before the start of the studied period. The chosen primary design resulted in an average follow-up time of 8.5 years. A longer time would have been an advantage, not only as it would have generated more data but also as the cohort would have been followed after the current maximum age of 24 years. Peak incidence is around 25 years for non-affective psychosis [44], and ~20 years for bipolar disorder [45]. However, in a performed sensitivity analysis no indications of trends or differences between age-spans within the current material were observed.

Possible confounding by acne was investigated in a sensitivity analysis. Exclusion of all individuals with a documented acne diagnosis resulted, for both non-affective psychosis and bipolar disorder, in no evidence for acne confounding the observed associations. An alternative approach would have been to control for acne diagnosis in the primary analysis. However, acne diagnosis was likely to be under-recorded as it only included hospital outpatients and inpatient records. It is therefore expected that patients with acne (not recorded in the current dataset) might not have been excluded from this sensitivity analysis. Also, to control for acne in the primary analysis would—in contrast to the chosen sensitivity analysis—not avoid potential confounding by more complex causal patterns related to acne, such as suggested by Jones et al. [28]. The result from the separate isotretinoin analysis, that did not indicate any substantial association between the nonantibiotic acne drug and SMI (the crude HR for non-affective psychosis was weakly significant observed in isolation, but did not provide evidence for an association given the total number of sensitivity analysis estimates), was a finding consistent with the null result for isotretinoin reported by Sellgren et al. [24].

In the main analyses, adjusted estimates were calculated using relevant covariates available from national registers. However, there may still be unmeasured or residual confounding from a number of sources. Aside from genetic predisposition for schizophrenia and bipolar disorder (only to a minor extent captured by the current covariates), overall healthcare usage was identified as being of potential importance. Healthcare utilization could be a link between bipolar disorder predisposition and antibiotic exposure, i.e., prodromal bipolar symptoms leads to overall ill health, higher healthcare utilization, and greater opportunity for antibiotic exposure. Unfortunately, a direct measurement of healthcare utilization was not obtainable on a full national level as the National Patient Register only covered specialized care. However, as the National Prescribed Drug Register provided full coverage from mid 2005, i.e., from both specialized and primary care, one way to view the exposure measurements of groups of antibiotics is as a rough proxy for overall healthcare utilization or, alternatively, as a proxy for overall health problems or propensity to seek healthcare for infections.

Another limitation is that the potential effects of minocycline could not be studied, as a result of minocycline not being prescribed in Sweden. Both minocycline and doxycycline have been reported to inhibit activation of microglial cells in different experimental models of neuroinflammation [20, 46, 47]. Whereas minocycline has specifically been reported to inhibit complement-mediated engulfment of synapses in vitro [24], such studies have, to the best of our knowledge, not been reported for doxycycline. Moreover, minocycline has been reported to reach higher levels in the brain as compared to doxycycline [21], which may explain the discrepancies between our findings and those of Sellgren et al. [24].

### Conclusions

There is no evidence in Swedish national registers for a preventive effect of doxycycline exposure during adolescence on the risk of subsequent non-affective psychosis diagnosis. Taken together with results of the recently reported add-on trial of minocycline [48], our present study suggest that minocycline or doxycycline are of little value for the prevention or treatment of schizophrenia.

There is evidence for an association between doxycycline exposure and increased risk of subsequent bipolar disorder diagnosis. However, similar associations were found for all other investigated groups of antibiotics and the relationship is therefore assumed to be explained by confounding or reverse causality.

## Supplementary information

Supplementary tables
