# Supplementary material for: Doxycycline exposure during adolescence and future risk of non-affective psychosis and bipolar disorder: a total population cohort study
Source: Transl Psychiatry. 2021 Sep 8;11:468. doi: 10.1038/s41398-021-01574-6 (PMC8426383; doi:10.1038/s41398-021-01574-6)
Supplement: Supplementary file 1 — Supplementary tables [file 41398_2021_1574_MOESM1_ESM.doc]

Supplementary Table 1. ATC-codes

|  | **ATC-code** |
| --- | --- |
| **Exposure** |
|  |  |
| **Antibiotics** |  |
| - Cefalosporines |  |
| Cefadroxil | J01DB05 |
|  |  |
| - Fluoroquinolones |  |
| Ciprofloxacin | J01MA02 |
| Levofloxacin | J01MA12 |
| Moxifloxacin | J01MA14 |
| Norfloxacin | J01MA06 |
|  |  |
| - Macrolides and lincosamides |  |
| Azithromycin | J01FA10 |
| Clarithromycin | J01FA09 |
| Clindamycin | J01FF01 |
| Erythromycin | J01FA01 |
| Roxithromycin | J01FA06 |
|  |  |
| - Nitrofurantoin |  |
| Nitrofurantoin | J01XE01 |
|  |  |
| - Penicillins |  |
| Amoxicillin | J01CA04 |
| Amoxicillin + enzyme inhibitor | J01CR02 |
| Flucloxacillin | J01CF05 |
| Phenoxymethylpenicillin [Penicillin V] | J01CE02 |
| Pivmecillinam | J01CA08 |
|  |  |
| - Tetracyclines |  |
| Doxycycline (primary exposure) | J01AA02 |
| Lymecycline | J01AA04 |
| Tetracycline | J01AA07 |
|  |  |
| - Trimethoprim |  |
| Trimethoprim | J01EA01 |
| Trimethoprim + sulfamethoxazol | J01EE01 |
|  |  |
| **Non antibiotics**  - Retinoids for treatment of acne |  |
| Isotretinoin | D10BA01 |
|  |  |
|  |  |

**Supplementary Table 2. Distribution of primary exposure across covariates (n=541 940)**

| **Factor** | **Level** | **Doxycycline 0–3 000 mg** | | **Doxycycline >3 000 mg** | | ***p*-value^1^** |
| --- | --- | --- | --- | --- | --- | --- |
| n |  | **534 897** |  | **7 043** |  |  |
| Sex | Female | **260 794** | (48.8%) | **3 833** | (54.4%) | *p*<0.001 |
|  | Male | **274 103** | (51.2%) | **3 210** | (45.6%) |  |
| Swedish healthcare region | Missing | **993** | (0.2%) | **0** | (0.0%) | *p*<0.001 |
|  | Stockholm | **111 538** | (20.9%) | **1 684** | (23.9%) |  |
|  | Uppsala | **19 321** | (3.6%) | **261** | (3.7%) |  |
|  | Södermanland | **15 965** | (3.0%) | **257** | (3.6%) |  |
|  | Östergötland | **25 260** | (4.7%) | **279** | (4.0%) |  |
|  | Jönköping | **20 870** | (3.9%) | **129** | (1.8%) |  |
|  | Kronoberg | **10 594** | (2.0%) | **118** | (1.7%) |  |
|  | Kalmar | **13 539** | (2.5%) | **157** | (2.2%) |  |
|  | Gotland | **3 385** | (0.6%) | **31** | (0.4%) |  |
|  | Blekinge | **8 337** | (1.6%) | **149** | (2.1%) |  |
|  | Skåne | **68 580** | (12.8%) | **897** | (12.7%) |  |
|  | Halland | **18 403** | (3.4%) | **245** | (3.5%) |  |
|  | Västra Götaland | **90 114** | (16.8%) | **1 249** | (17.7%) |  |
|  | Värmland | **15 685** | (2.9%) | **200** | (2.8%) |  |
|  | Örebro | **16 397** | (3.1%) | **208** | (3.0%) |  |
|  | Västmanland | **14 716** | (2.8%) | **198** | (2.8%) |  |
|  | Dalarna | **16 005** | (3.0%) | **182** | (2.6%) |  |
|  | Gävleborg | **15 556** | (2.9%) | **198** | (2.8%) |  |
|  | Västernorrland | **13 539** | (2.5%) | **188** | (2.7%) |  |
|  | Jämtland | **7 179** | (1.3%) | **73** | (1.0%) |  |
|  | Västerbotten | **14 858** | (2.8%) | **161** | (2.3%) |  |
|  | Norrbotten | **14 063** | (2.6%) | **179** | (2.5%) |  |
| Origin of the individual | Sweden | **495 844** | (92.7%) | **6 739** | (95.7%) | *p*<0.001 |
|  | South and Central America | **2 361** | (0.4%) | **18** | (0.3%) |  |
|  | Eastern Europe, Baltics and Russia | **4 504** | (0.8%) | **29** | (0.4%) |  |
|  | Sub-Saharan Africa | **3 657** | (0.7%) | **21** | (0.3%) |  |
|  | Other international region | **28 446** | (5.3%) | **235** | (3.3%) |  |
|  | Unknown | **85** | (<.1%) | **1** | (<.1%) |  |
| Origin of parents | Sweden | **387 873** | (72.5%) | **5 458** | (77.5%) | *p*<0.001 |
|  | One parent not from Sweden | **58 186** | (10.9%) | **807** | (11.5%) |  |
|  | Both parents not from Sweden | **88 838** | (16.6%) | **778** | (11.0%) |  |
| Income of parents | Missing | **5 644** | (1.1%) | **49** | (0.7%) | *p*<0.001 |
|  | Lowest (1) | **115 932** | (21.7%) | **1 228** | (17.4%) |  |
|  | 2 | **122 310** | (22.9%) | **1 499** | (21.3%) |  |
|  | 3 | **121 566** | (22.7%) | **1 680** | (23.9%) |  |
|  | 4 | **85 348** | (16.0%) | **1 302** | (18.5%) |  |
|  | Highest (5) | **84 097** | (15.7%) | **1 285** | (18.2%) |  |
| Highest completed education among parents | Missing | **141 621** | (26.5%) | **1 730** | (24.6%) | *p*<0.001 |
|  | Less than elementary school | **3 996** | (0.7%) | **23** | (0.3%) |  |
|  | Elementary school | **11 399** | (2.1%) | **160** | (2.3%) |  |
|  | Upper secondary school <2 years | **103 216** | (19.3%) | **1 383** | (19.6%) |  |
|  | Upper secondary school 3 years | **79 793** | (14.9%) | **1 022** | (14.5%) |  |
|  | Post-secondary education <3 years | **78 365** | (14.7%) | **1 073** | (15.2%) |  |
|  | Post-secondary education >3 years | **108 315** | (20.2%) | **1 501** | (21.3%) |  |
|  | Postgraduate education | **8 192** | (1.5%) | **151** | (2.1%) |  |
| One or both parents diagnosed with non-affective psychosis | No | **526 635** | (98.5%) | **6 934** | (98.5%) | *p*=0.980 |
|  | Yes | **8 262** | (1.5%) | **109** | (1.5%) |  |
| One or both parents diagnosed with bipolar disorder | No | **525 516** | (98.2%) | **6 890** | (97.8%) | *p*=0.008 |
|  | Yes | **9 381** | (1.8%) | **153** | (2.2%) |  |

^1^ *p*-values represent the support for an unequal distribution across the covariate categories

**Supplementary Table 3. Distribution of non-affective psychosis across covariates (n=541 940)**

| **Factor** | **Level** | **No F20–F29 diagnosis during follow-up** | | **First diagnosis of F20–F29 during follow-up** | | ***p*-value^1^** |
| --- | --- | --- | --- | --- | --- | --- |
| n |  | **539 758** |  | **2 182** |  |  |
| Sex | Female | **263 784** | (48.9%) | **843** | (38.6%) | *p*<0.001 |
|  | Male | **275 974** | (51.1%) | **1 339** | (61.4%) |  |
| Swedish healthcare region | Missing | **992** | (0.2%) | **1** | (<.1%) | *p*<0.001 |
|  | Stockholm | **112 699** | (20.9%) | **523** | (24.0%) |  |
|  | Uppsala | **19 487** | (3.6%) | **95** | (4.4%) |  |
|  | Södermanland | **16 149** | (3.0%) | **73** | (3.3%) |  |
|  | Östergötland | **25 445** | (4.7%) | **94** | (4.3%) |  |
|  | Jönköping | **20 900** | (3.9%) | **99** | (4.5%) |  |
|  | Kronoberg | **10 687** | (2.0%) | **25** | (1.1%) |  |
|  | Kalmar | **13 635** | (2.5%) | **61** | (2.8%) |  |
|  | Gotland | **3 404** | (0.6%) | **12** | (0.5%) |  |
|  | Blekinge | **8 450** | (1.6%) | **36** | (1.6%) |  |
|  | Skåne | **69 225** | (12.8%) | **252** | (11.5%) |  |
|  | Halland | **18 612** | (3.4%) | **36** | (1.6%) |  |
|  | Västra Götaland | **91 021** | (16.9%) | **342** | (15.7%) |  |
|  | Värmland | **15 820** | (2.9%) | **65** | (3.0%) |  |
|  | Örebro | **16 541** | (3.1%) | **64** | (2.9%) |  |
|  | Västmanland | **14 853** | (2.8%) | **61** | (2.8%) |  |
|  | Dalarna | **16 124** | (3.0%) | **63** | (2.9%) |  |
|  | Gävleborg | **15 692** | (2.9%) | **62** | (2.8%) |  |
|  | Västernorrland | **13 661** | (2.5%) | **66** | (3.0%) |  |
|  | Jämtland | **7 228** | (1.3%) | **24** | (1.1%) |  |
|  | Västerbotten | **14 968** | (2.8%) | **51** | (2.3%) |  |
|  | Norrbotten | **14 165** | (2.6%) | **77** | (3.5%) |  |
| Origin of the individual | Sweden | **500 650** | (92.8%) | **1 933** | (88.6%) | *p*<0.001 |
|  | South and Central America | **2 358** | (0.4%) | **21** | (1.0%) |  |
|  | Eastern Europe, Baltics and Russia | **4 494** | (0.8%) | **39** | (1.8%) |  |
|  | Sub-Saharan Africa | **3 622** | (0.7%) | **56** | (2.6%) |  |
|  | Other international region | **28 549** | (5.3%) | **132** | (6.0%) |  |
|  | Unknown | **85** | (<.1%) | **1** | (<.1%) |  |
| Origin of parents | Sweden | **392 038** | (72.6%) | **1 293** | (59.3%) | *p*<0.001 |
|  | One parent not from Sweden | **58 664** | (10.9%) | **329** | (15.1%) |  |
|  | Both parents not from Sweden | **89 056** | (16.6%) | **560** | (25.7%) |  |
| Income of parents | Missing | **5 645** | (1.0%) | **48** | (2.2%) | *p*<0.001 |
|  | Lowest (1) | **116 528** | (21.6%) | **632** | (29.0%) |  |
|  | 2 | **123 340** | (22.9%) | **469** | (21.5%) |  |
|  | 3 | **122 809** | (22.8%) | **437** | (20.0%) |  |
|  | 4 | **86 374** | (16.0%) | **276** | (12.6%) |  |
|  | Highest (5) | **85 062** | (15.8%) | **320** | (14.7%) |  |
| Highest completed education among parents | Missing | **142 691** | (26.4%) | **660** | (30.2%) | *p*<0.001 |
|  | Less than elementary school | **3 994** | (0.7%) | **25** | (1.1%) |  |
|  | Elementary school | **11 490** | (2.1%) | **69** | (3.2%) |  |
|  | Upper secondary school <2 years | **104 165** | (19.3%) | **434** | (19.9%) |  |
|  | Upper secondary school 3 years | **80 557** | (14.9%) | **258** | (11.8%) |  |
|  | Post-secondary education <3 years | **79 155** | (14.7%) | **283** | (13.0%) |  |
|  | Post-secondary education >3 years | **109 407** | (20.3%) | **409** | (18.7%) |  |
|  | Postgraduate education | **8 299** | (1.5%) | **44** | (2.0%) |  |
| One or both parents diagnosed with non-affective psychosis | No | **531 539** | (98.5%) | **2 030** | (93.0%) | *p*<0.001 |
|  | Yes | **8 219** | (1.5%) | **152** | (7.0%) |  |
| One or both parents diagnosed with bipolar disorder | No | **530 307** | (98.2%) | **2 099** | (96.2%) | *p*<0.001 |
|  | Yes | **9 451** | (1.8%) | **83** | (3.8%) |  |

^1^ *p*-values represent the support for an unequal distribution across the covariate categories

**Supplementary Table 4. Distribution of bipolar disorder across covariates (n=541 940)**

| **Factor** | **Level** | **No F30–F31 diagnosis during follow-up** | | **First diagnosis of F30–F31 during follow-up** | | ***p*-value^1^** |
| --- | --- | --- | --- | --- | --- | --- |
| n |  | **538 573** |  | **3 367** |  |  |
| Sex | Female | **262 237** | (48.7%) | **2 390** | (71.0%) | *p*<0.001 |
|  | Male | **276 336** | (51.3%) | **977** | (29.0%) |  |
| Swedish healthcare region | Missing | **993** | (0.2%) | **0** | (0.0%) | *p*<0.001 |
|  | Stockholm | **112 480** | (20.9%) | **742** | (22.0%) |  |
|  | Uppsala | **19 361** | (3.6%) | **221** | (6.6%) |  |
|  | Södermanland | **16 098** | (3.0%) | **124** | (3.7%) |  |
|  | Östergötland | **25 411** | (4.7%) | **128** | (3.8%) |  |
|  | Jönköping | **20 902** | (3.9%) | **97** | (2.9%) |  |
|  | Kronoberg | **10 659** | (2.0%) | **53** | (1.6%) |  |
|  | Kalmar | **13 624** | (2.5%) | **72** | (2.1%) |  |
|  | Gotland | **3 381** | (0.6%) | **35** | (1.0%) |  |
|  | Blekinge | **8 458** | (1.6%) | **28** | (0.8%) |  |
|  | Skåne | **69 209** | (12.9%) | **268** | (8.0%) |  |
|  | Halland | **18 551** | (3.4%) | **97** | (2.9%) |  |
|  | Västra Götaland | **90 851** | (16.9%) | **512** | (15.2%) |  |
|  | Värmland | **15 785** | (2.9%) | **100** | (3.0%) |  |
|  | Örebro | **16 531** | (3.1%) | **74** | (2.2%) |  |
|  | Västmanland | **14 784** | (2.7%) | **130** | (3.9%) |  |
|  | Dalarna | **16 058** | (3.0%) | **129** | (3.8%) |  |
|  | Gävleborg | **15 569** | (2.9%) | **185** | (5.5%) |  |
|  | Västernorrland | **13 644** | (2.5%) | **83** | (2.5%) |  |
|  | Jämtland | **7 224** | (1.3%) | **28** | (0.8%) |  |
|  | Västerbotten | **14 915** | (2.8%) | **104** | (3.1%) |  |
|  | Norrbotten | **14 085** | (2.6%) | **157** | (4.7%) |  |
| Origin of the individual | Sweden | **499 379** | (92.7%) | **3 204** | (95.2%) | *p*<0.001 |
|  | South and Central America | **2 361** | (0.4%) | **18** | (0.5%) |  |
|  | Eastern Europe, Baltics and Russia | **4 506** | (0.8%) | **27** | (0.8%) |  |
|  | Sub-Saharan Africa | **3 660** | (0.7%) | **18** | (0.5%) |  |
|  | Other international region | **28 581** | (5.3%) | **100** | (3.0%) |  |
|  | Unknown | **86** | (<.1%) | **0** | (0.0%) |  |
| Origin of parents | Sweden | **390 857** | (72.6%) | **2 474** | (73.5%) | *p*<0.001 |
|  | One parent not from Sweden | **58 439** | (10.9%) | **554** | (16.5%) |  |
|  | Both parents not from Sweden | **89 277** | (16.6%) | **339** | (10.1%) |  |
| Income of parents | Missing | **5 644** | (1.0%) | **49** | (1.5%) | *p*=0.025 |
|  | Lowest (1) | **116 483** | (21.6%) | **677** | (20.1%) |  |
|  | 2 | **123 065** | (22.9%) | **744** | (22.1%) |  |
|  | 3 | **122 470** | (22.7%) | **776** | (23.0%) |  |
|  | 4 | **86 075** | (16.0%) | **575** | (17.1%) |  |
|  | Highest (5) | **84 836** | (15.8%) | **546** | (16.2%) |  |
| Highest completed education among parents | Missing | **142 497** | (26.5%) | **854** | (25.4%) | *p*=0.001 |
|  | Less than elementary school | **4 008** | (0.7%) | **11** | (0.3%) |  |
|  | Elementary school | **11 473** | (2.1%) | **86** | (2.6%) |  |
|  | Upper secondary school <2 years | **103 880** | (19.3%) | **719** | (21.4%) |  |
|  | Upper secondary school 3 years | **80 323** | (14.9%) | **492** | (14.6%) |  |
|  | Post-secondary education <3 years | **78 981** | (14.7%) | **457** | (13.6%) |  |
|  | Post-secondary education >3 years | **109 127** | (20.3%) | **689** | (20.5%) |  |
|  | Postgraduate education | **8 284** | (1.5%) | **59** | (1.8%) |  |
| One or both parents diagnosed with non-affective psychosis | No | **530 342** | (98.5%) | **3 227** | (95.8%) | *p*<0.001 |
|  | Yes | **8 231** | (1.5%) | **140** | (4.2%) |  |
| One or both parents diagnosed with bipolar disorder | No | **529 425** | (98.3%) | **2 981** | (88.5%) | *p*<0.001 |
|  | Yes | **9 148** | (1.7%) | **386** | (11.5%) |  |

^1^ *p*-values represent the support for an unequal distribution across the covariate categories
